# Perceived Risk Factors for Suicide among Nepalese Migrant Workers in South Korea

**DOI:** 10.3390/ijerph18126368

**Published:** 2021-06-11

**Authors:** Madhu Sudhan Atteraya, Nasser B. Ebrahim, Shreejana Gnawali

**Affiliations:** 1 Department of Social Welfare, Keimyung University, Daegu 42601, Korea; 2 Department of Public Health, Keimyung University, Daegu 42601, Korea; nasser.ebrahim9@gmail.com; 3 Department of Sociology, The Academy of Korean Studies (AKS), Seongnam-si 03172, Korea; shreejana@hanmail.net

**Keywords:** migrant workers, suicide, risk factors

## Abstract

(1) Background: In South Korea, far from their homeland, Nepalese migrant workers often face tremendous challenges. The most severe outcome for migrant workers is death by suicide—a major cause of premature death among migrant workers. Nevertheless, in the literature, key factors associated with suicide among Nepalese migrant workers are not specifically identified. Thus, we aimed to delineate the main suicide risk factors for this group of migrants. (2) Methods: We used qualitative research methodology (sample = 20; male =17, female = 3) and employed nominal group techniques to identify the perceived primary risk factors for suicide. (3) Results: Study participants identified and ranked eight sources of distress and perceived risks for suicide, both from home and in the host country. Perceived risks for suicide include a complex set of socio-cultural, behavioral, occupational, physical, and mental health issues as well as communication barriers. (4) Conclusions: The findings suggest the need to design tailored mental health promotion programs for migrant workers before departure from Nepal as well as after arrival as migrant workers in South Korea.

## 1. Introduction

Worldwide, suicide is a significant public health and social welfare problem. It is one of the leading causes of mortality in adults, resulting globally in the death of 800,000 people each year [1]. Although there is a dearth of quality data regarding suicide among migrant workers [1], recent studies have shown a prevalence of mental health problems, suicidal ideation, and attempts among migrant workers from low-income countries [2,3,4,5].

Understanding the suicide risk among migrants is complex, as suicidal ideation and/or suicide attempts are influenced by a myriad of socio-cultural and economic factors in both the country of origin and destination, including demographic factors such as age and gender and mental health problems including depression, anxiety, and hopelessness [6,7,8,9,10,11,12,13,14]. Migrant workers as a vulnerable group also experience social exclusion and mental health issues in host countries, which further lead to higher risk for suicide ideation and attempt [12,15].

Evidence from the literature suggests that immigrant status is positively associated with suicide ideation and attempt [6,7,8,9,10,11,12,13,14,15]. For example, a study that investigated attempted suicides among Bhutanese refugees in the United States reported that suicide risk includes, among other factors, a lack of social welfare support (e.g., employment and resettlement difficulties, inadequate social support, family separation and frustration, and integration difficulties) [14]. Similarly, female Mexican farmworkers in the U.S. were reported to disproportionately suffer from anxiety, depression, low self-esteem, family dysfunction, ineffective social support, and suicide ideation [6]. Correspondingly, suicide risk factors among migrant workers in the United Arab Emirates were reported to be associated with physical illness, lower level of wages, long working hours, and depression [7]. Likewise, in a study that involved 78 Filipino home care workers in Israel, the perceived level of discrimination and abuse were specified as risk factors for suicide [8]. Evidence from Australia also showed that immigrants disproportionately commit suicide, with more than 25% of suicides committed by immigrants [9]. Similarly, in England, Israel, and the Netherlands higher suicide rates were reported among immigrant communities [10].

A comprehensive understanding of key risk factors for suicide and suicide attempts and implementing appropriate risk reduction strategies are the first and most important strategies to prevent suicide [15,16,17]. As such, Joiner [17] delineated the risk factors for suicide, which may include a combination of social isolation, loneliness, family separation, lack of social connection, hopelessness, physical and mental illness, and the opportunity to engage in risky behavior. Furthermore, preventive approaches to reduce suicide may include conducting population-based surveillance as well as identifying vulnerable populations and the issues related to their health/mental health status. However, migrant workers—specifically those coming from low-income countries—are among the most vulnerable populations, and conducting mental health surveillance before international migration is nonexistent in most of developing countries, including Nepal [15]. It is also a challenge in the host countries, partly due to the transitory nature of migrant employment.

### 1.1. South Korean Context

South Korea’s remarkable economic success and improved living standards have created a labor shortage in the so-called 3D (dangerous, dirty, and difficult) jobs available in small and medium sized enterprises (SMEs) [18]. To address this shortage, the South Korean government has allowed companies to recruit migrant workers [19]. Through the Employment Permit System (EPS), migrant workers from 16 countries (Vietnam, Philippines, Thailand, Indonesia, Mongolia, Sri Lanka, China, Uzbekistan, Pakistan, Cambodia, Nepal, Myanmar, Kyrgyzstan, Bangladesh, East Timor, and Laos) are allowed to work temporarily in Korean companies. As a result, there are now about 260,000 migrant workers under the EPS system, which account for 21% of all foreigners living in the country [20]. Due to a massive demand for labor in SMEs, the number of migrant workers is expected to rise in the coming years.

For migrant workers, employment in South Korea has an economic advantage, as they can earn much higher wages than what they would have earned in their home countries—a significant pull factor for foreign migrant workers. However, in the deeply hierarchical social structure of Korean society, migrant workers occupy the lowest position in the pyramid of social class. Thus, less emphasis is placed on social and cultural integration, as migrant worker’s issues are primarily seen through the prism of economic contribution and a source of cheap labor [21]. Many studies have documented the acculturation and adaptation difficulties faced by migrant workers, along with work site injuries and mental health problems such as depression and suicide ideation and attempt [22,23,24,25,26].

### 1.2. Deaths among Nepalese Migrant Workers in South Korea

The number of Nepalese migrant workers arriving in South Korea has increased rapidly. In the year 2000, there were only 1727 Nepalese migrant workers; the number jumped to 35,136 by mid-2019, making Nepal the third largest source of migrant workers after Vietnam (37,059) and Cambodia (37,880) [27]. The statistics from the Ministry of Justice [27] suggest a steady increase of Nepalese migrant workers in South Korea since 2000. As the number of migrant workers increased, so did the deaths among Nepalese migrant workers in South Korea [28]. For example, from 2007 to April 2019, there were 170 deaths reported among Nepalese migrant workers [29,30]. Seventy-six (44.7%) were from natural causes, 47 (27.6%) from suicide, 45 (26.5%) from accidents and 2 (1.2%) were homicides. Among the tragedies involving Nepalese migrant workers, one case that was highly discussed in the Korean and international media was the suicide of a Nepalese migrant worker on 7 August 2017, who left a short note before taking his own life [31]. The direct translation read “Hello, everyone. I am saying goodbye to the world today. I’ve had health problems and I can’t sleep. I’ve been receiving treatment, but haven’t gotten better. It’s been so difficult for me, and today I have decided to leave this world. I’ve had to deal with stress at work and want to go to another factory, but to no avail. Even though I’ve tried to go to Nepal for treatment, I haven’t been able to do so because the company did not allow me” [31]. This tragedy signifies the vulnerabilities of migrant workers to suicide and the need to identify suicide risk factors. Thus, the purpose of this study was explore and delineate the main suicide risk factors among this group of migrant workers.

## 2. Materials and Methods

### 2.1. Research Participants

The objective of this exploratory research was to identify the key contributing risk factors for suicide among Nepalese migrant workers in South Korea. For this purpose, we assembled a multidisciplinary research team from social welfare, sociology, and public health disciplines. The team conducted a series of meetings, putting in place strategies and plans, including research design, participant recruitment, research location, and other logistical issues. Subsequently, the research team reached out to the Nepalese self-help communities (Daegu Nepalese Shelter for Migrant Workers and two self-help groups of migrant communities—Daegu Magar Sanga and Sherpa Samaj), who showed interest in the research theme and agreed to provide support. Accordingly, the Nepalese migrant workers’ self-help communities helped with study participant recruitment (i.e., Daegu Nepalese Shelter for Migrant Workers, Daegu Magar Sanga and Sherpa Samaj). The criteria were that participants be male or female, either a former or current migrant worker, 18 years or older, living in South Korea for at least six months, and voluntarily consenting to participate in the study. The research protocol and data collection methods were reviewed and approved by the Institutional Review Board of Keimyung University (IRB No. 40525-201707-HR-52-01). In total, 20 participants were recruited for the study (male = 17, female = 3), with diverse backgrounds including migrant workers and community leaders (See Table 1).

### 2.2. Research Design

Study participants took part in two consecutive meetings at Daegu Migrant Center, each lasting three hours. The meeting was conducted in the Nepali language. In the first meeting, participants were introduced to the research team, which was followed by a presentation on the significance, objectives, and planned activities, including registration and completion of consent forms. Participants were also introduced to the nominal group process activities and familiarized with the planned data collection methods.

The following week, we conducted the second meeting in the same venue and used the nominal group technique (NGT) to collect data. NGT gathers data by asking research participants to respond to questions posed by a moderator and then asks participants to rank the ideas or suggestions of all group members. Pens and several small square papers were distributed to the research participants. The moderator posted on the whiteboard the main question: “Why do you think a migrant worker would commit suicide here in Korea?” Each participant in the study was asked to give five important reasons. At this stage, no discussion was allowed, because we wanted to tease out diverse ideas from the group. After we made sure that every participant had written their five answers, an assistant to the moderator collected all of the participants’ responses. Next, the group’s answers were sorted according to themes, with redundant ideas dropped with agreement from the originator of the idea. Then, a group discussion ensued to rank the emerging themes. Notes were taken during the discussion as well. Rankings of the perceived reasons contributing to suicide among Nepalese migrants were finally specified by the group through unanimous agreement. Lunch and refreshments were served, and participants were thanked for their participation. Results were finally translated from Nepali to English and back- translated to Nepali to ensure language meaning equivalencies.

We, the researchers, are also foreign residents (including Nepalese citizens) in South Korea and are aware of the difficulties and challenges faced by migrant workers. However, we lack complete understanding of what is like to be a migrant worker and their intimate experiences of daily challenges. Although we have our own perspectives in this regard, we strived to tell their stories and experiences.

## 3. Results

We asked study participants the question, “Why do you think a migrant worker would commit suicide here in Korea?” The purpose was to identify and rank perceived reasons for suicide among Nepalese migrant workers in South Korea. Correspondingly, study participants identified the following eight perceived risks and sources of distress in the country of origin as well as in the host country: (1) family issues in Nepal, (2) loose camaraderie at work, (3) drudgery and hazardous work, (4) family nescience of migrant life, (5) inter-migrant competition, (6) addiction to gambling, (7) physical and mental health issues, and (8) adaptation problems and communication barriers. These risk factors as evidenced by study participants are presented in order of their importance.

### 3.1. Family Issues in Nepal

Study participants identified family issues back home (Nepal) as the most significant risk factor and the main source of distress among migrant works. Family issues arose from conflicts between spouses and between spouses and in-laws, misunderstandings, suspicions—especially about extra-marital relations, higher monetary expectations, and so on.

“Generally, Nepalese men get married at the age 25. I got married when I was 23 years old. While I was in Nepal, I did not have any problems. Now, I often receive phone calls from my wife about conflicts with my parents. Yesterday, I received a call from my wife; she was complaining about my mother. This gives me a lot of stress.”

(Worker 2)

A community leader added: Suspicion and mistrust are also significant reasons for family conflicts.

“Generally, the easy accessibility of the internet in Korea, and by extension, social media dating platforms create uneasiness among wives in Nepal who live far away from their spouses, i.e., young males living alone. This creates suspicions, conflict, and discord in families that further contribute to the stress among migrant workers.”

(Community Leader and Worker 1)

Another community leader elaborated on how family members start business investments in Nepal (such as buying houses or land) on behalf of migrant workers without knowing their financial status.

“Without knowing the working conditions and the level of income in Korea, migrants’ families in Nepal engage in investment planning shortly after the migrant’s arrival in Korea. Some families initiate small businesses or buy land in urban areas to build a house or even start construction without knowing our income. In general, the perception about migrant workers in Nepal is that we make a lot of money.”

(Community Leader and Worker 2)

In addition, migrant family members in Nepal expect economic benefits from migrant workers and become unhappy when the benefits fall short of their expectations, especially in comparison with other families.

“Some of the parents exaggerate their son’s/daughter’s wages to the neighbors and relatives, and parents who have heard this, but haven’t received the expected amount of money from their sons/daughters, start to complain. Oblivious to the situation in Korea, these families assume that their sons/daughters are not earning enough or they are spending money on unnecessary things. These suspicions and over-expectations of parents are also sources of tension and conflict in families. These are the reason for developing depression among migrant workers in South Korea.”

(Community Leader and Worker 3)

Migrant workers also made the following remarks about family issues from Nepal:

“My sister-in-law bought land in Kathmandu; what have you done?”

(Worker 6)

“My friend bought a big necklace at this festival; I want a bigger one. Send me money.”

(Worker 7)

“I can’t stay with your parents; rent a house for me in the city.”

(Worker 6)

### 3.2. Loose Camaraderie at Work

A lack of camaraderie at work is an important source of distress and perceived risk for suicide among Nepalese migrant workers in South Korea, as reflected by study participants. The loose camaraderie and lack of mutual support in the workplace are evidenced by their comments.

In general, Nepalese migrant workers live and work harmoniously in South Korea. However, there are cases where a senior Nepalese migrant worker’s overbearing behavior toward fellow Nepalese workers creates uneasiness and stress at work. Some junior Nepalese workers receive pressure from senior Nepalese workers at work, which in and of itself is a source of stress. It is a common behavior to curry favor with the Korean managers as a signal to the senior managers (Korean) that a worker is managing tasks properly. Often, senior Nepalese workers do not offer support and even criticize junior workers in the presence of higher management (as mentioned by Community Leader and Worker 2).

A migrant worker shared his experience in this regard and noted the following:

“My senior Nepalese manager told me that I will be fired soon. Distraught, I talked to the upper management who told me that I will not be fired. I reported him to the Human Resource Department and he was later fired.”

(Worker 15)

Even though caste and ethnicity-based discrimination was legally abolished in Nepal, some Nepalese migrant workers still adhere to the customs and practices of untouchability and caste-based discrimination while in Korea, which is a source of alienation and distress.

“I know that in the dormitory inside the Seonse Industrial Complex, some of the high Hindu caste factory workers practice untouchability of food and water by lower castes. Moreover, they offer help to people from their caste or ethnicity and are reluctant to help or share information with others.”

(Community Leader 3)

“On one occasion, I recommended one Brahmin friend to work in a company, where most workers were from Magar (a kind of indigenous group); all were angry with me.”

(Community Leader and Worker 3)

### 3.3. Drudgery and Hazardous Work

The hazardous work and long working hours are also risk factors for physical and mental health problems, as evidenced by the following comments from migrant workers.

“We are working in the 3D (dangerous, dirty, and difficult) jobs. Work is too hard. I now have back pain and am living in a shelter.”

(Worker 16)

“For those who work in the agricultural sector, the working hours are very long. It causes physical and mental health problems.”

(Community Leader and Worker 3)

“Work is very hazardous and difficult. It involves melting iron, die-casting, and working with various toxic chemicals. It is challenging to work all day long.”

(Worker 14)

### 3.4. Family Nescience of Migrant Life

Most migrant families living in Nepal lack adequate knowledge of the challenges in Korea and only emphasize the economic benefits, and this can put migrant workers under pressure. To this point, a migrant and community leader said the following:

“Nepalese media and people only emphasize higher wages in Korea. But they fail to consider the challenges of working in Korea. The challenges of working in Korea became apparent just after we started working here. We later realized how difficult the work is and how many hours we have to work to earn a living. The gap between our expectations and the reality is very wide.”

(Community Leader and Worker 3)

### 3.5. Addiction to Gambling

Based on the discussion with community leaders, migrant workers’ addiction to gambling is an emerging problem. Migrants who are addicted to gambling often lose their hard-earned money, putting them under financial stress, with severe mental health consequences, as evidenced by the following comments.

“These days, some migrant workers are addicted to casinos and gambling. First, they play for curiosity and experiment with a little amount. Fooled by a few wins, the temptation grows, and before they know it, they find themselves addicted to gambling and lose money. Still, they do not stop playing in the hope that someday they will get their money back. So they borrow money from friends and go to the casinos again. They again lose all the money. Do you know Alex? (not real name). He lost all his money. He disappeared and nobody knows where he is now.”

(Community Leader and Worker 3)

“Now you feel that you are in between the two blades of a scissor. You’ve lost all your money and don’t have any and you are also obliged to return the borrowed money to a friend. At the same time, back home your family is waiting for your money. To escape the mounting pressure, many addicted gamblers commit suicide.”

(Community Leader and Worker 2)

### 3.6. Inter-Migrant Competition

Income competition among migrant workers could also be a source of distress.

“Migrant workers compare their wages among themselves and aim to attain higher earnings similar to other migrant workers who make more money. Some of the workers tend to change jobs; however, some fail to secure a new position in time and become undocumented and risk deportation.”

(Community Leader and Worker 1)

### 3.7. Poor Physical and Mental Health

Migrant workers often develop physical and mental health problems.

“Isolation, loneliness, and back pain from long hours of hard work are common. As a result, many migrant workers develop physical and mental health issues. Later, they develop anxiety and depression.”

(Community Leader and Worker 1)

### 3.8. Adaptation Problems and Communication Barriers

Adapting to the Korean way of life, including food and language barriers, can be a source of distress. Communication barriers can also constrain health service utilization.

“In the beginning, I had a very hard time finding food I liked. I felt so weak at work. Work was also very hard. I do not eat beef and pork and most Korean foods contain pork. I ate only rice and water for months.”

(Worker 13)

“Language is the biggest barrier for migrant workers in the workplace.”

(Community Leader and Worker 2)

“When we go to the hospital, it is hard to explain our symptoms and health problems. We cannot express our health problems, and doctors do not understand what we say.”

(Community Leader and Worker 3)

## 4. Discussion

Suicidal behavior is complex; however, an understanding of key risk factors can be an avenue to explore the underlying reasons that further help prevent suicide outcomes [17,32]. The current study explores the suicide risk factors among Nepalese migrant workers in South Korea.

Since the Employment Permit System (EPS) was adopted by South Korea, the country has become one of the most attractive destinations for Nepalese migrant workers. As a result, by the end of March 2019, there were 40,220 Nepalese migrant workers in South Korea. Parallel to this, from 2007 to 2019, of the 170 recorded deaths among migrant workers, 47 (27.6%) were caused by suicide [29,30]. Although, it is difficult to pinpoint what triggers suicide among Nepalese migrant workers, study participants identified a complex set of perceived risk factors in the migrant sending country (Nepal) and the migrant receiving country (South Korea). Sources of distress include migrant families, suboptimal social relations, worksite problems, competition among migrants, addiction to gambling, and cultural and linguistic barriers.

Family issues in Nepal are one of the most important sources of distress for migrant workers. For example, married migrant men come to Korea for work unaccompanied, leaving their children and wives behind. During this time, wives may live with their mothers-in-law and are obliged to be subordinate. This may create conflict in the family, distressing migrant workers abroad. In addition, family separation was reported to exacerbate psychological problems [33]. Issues related to childbearing, disrupted family life, marital mistrust, and infidelity may further strain family relations and impact the psychological well-being of migrant workers [34].

Migration in and of itself is a disrupter of social relations, which are important for physical as well as mental well-being [35]. Study participants reported an erosion of social and family relations—an important resource for coping with stress [36]. For example, caste- and ethnicity-based discrimination and the practice of untouchability were abolished in Nepal; however, surprisingly, testimonies of the study participants suggest that a few previously untouchable castes (e.g., Dalits) experience untouchability of water and food in factory dormitories in Korea. This may alienate a segment of the migrant population, risking the “double ostracization” of being a migrant worker and being shunned by their fellow Nepalese. Further study is necessary to explore the magnitude of caste/ethnic discrimination in worksite settings and its contribution to mental health problems among migrant workers.

Although migrant workers are paid better wages in Korea than in their home countries, they often work in industries that demand hard work for long hours, causing injuries and even death. For example, from 2007 to 2019, 45 migrants died in worksite-related accidents [29,30]. Many also suffer physical injuries and chronic pain, affecting their mental well-being. Furthermore, job security is a constant irritant and a source of distress among many migrants. For example, switching employers, while permitted, has some consequences because work permits for migrant workers last for almost five years, after which time, migrant workers must return to their home countries. Those who stay with their original employer for the entire time can be rehired for a second period after three months of hiatus; however, those who switch employers during their first turn may lose this privilege, and if they want to be rehired, they must undergo a lengthy and arduous process. Thus, workers may choose to stay with the same company even under difficult circumstances. Switching work places also entails the risk of losing their working permit if a replacement job is not found within three months. Some workers who find themselves in this predicament become homeless and undocumented. Moreover, a top-down hierarchy in the workplace creates a pecking-order style of management, especially among Nepalese senior managers, that fosters fear and control among new arrivals. This was mentioned by study participants as one of the main sources of stress.

Migrant families as well as government and national media often emphasize the financial benefits of labor migration and its role in decreasing household poverty due to the increased remittance [37]. However, the negative externalities attributed to labor migration, such as family separation, divorce, and the psychological toll of migration, are often ignored [10,38,39]. Moreover, the lack of understanding of challenges faced by migrant workers and the ever-increasing migrant families’ demand for increased financial support, which in part is linked to the “demonstration effect” and consumerism, increases the pressure and stress on migrant workers. The pressure and stress among some migrants may lead to maladaptive behaviors such as addiction to alcohol and gambling.

Another important source of distress mentioned by study participants was the acculturation difficulties rooted in cultural differences and communication barriers [40,41,42]. These factors may negatively influence healthcare utilization for migrants, often forcing migrants to confront unsurmountable problems by themselves; repeated frustrations may lead to distress and mental health problems.

While these disparate sources of distress, experienced separately and occasionally, may not necessarily trigger suicide, collective and chronic exposure to stressors may lead to mental health problems, a risk factor for mental illness and suicide. In general, our findings may be explained in part by the interpersonal theory of suicide [17,32]. For example, social isolation along with family conflict, marital separation, loneliness, lack of mutual support in the work setting, and other social and work-related daily stressors may expose workers to mental health issues that may affect their work performance. Losing jobs and a family’s source of income are frowned upon among Nepalese people. Such failure to use the economic opportunities presented to them could be humiliating enough for the migrant workers to trigger suicidal thoughts and actions. Thus, initiating concrete suicide prevention intervention is important both in the migrant sending and receiving countries; this may include pre-departure training [15] and post-departure counselling and acculturation support. Programs that provide counseling before departure, mental health screening, accessibility to mental health services, and programs for family visitation may help foster mental well-being among Nepalese migrant workers.

### Limitations

The study was a small qualitative study; hence, the results are not generalizable beyond the study participants. Participation in the study was voluntary and thus was not fully representative of migrant workers and may not have captured a range of views about suicide risk factors among Nepalese migrant workers. However, we believe the results fully represent the views and opinions of the study participants. The study was exploratory, and the results should be confirmed in larger representative populations.

## 5. Conclusions

Study participants identified important risk factors for suicide among Nepalese migrant workers in South Korea. These include socio-cultural, behavioral, occupational, and physical and mental health issues as well as acculturation and communication barriers. The findings suggest the need to design tailored mental health promotion programs for migrant workers before departure from Nepal as well as after arrival in South Korea.

## Figures and Tables

**Table 1:** Demographic characteristics of the participants (*n* = 20).

Participants	Age	Gender	Job Type	Length of Stay
Worker_1	30	Male	Manufacturing	5 years
Worker_2	25	Male	Manufacturing	2 years
Worker_3	31	Male	Garment	9 years
Worker_4	26	Male	Agriculture	2 years
Worker_5	23	Male	Construction	3 years
Worker_6	29	Male	Construction	6 years
Worker_7	34	Male	Furniture	8 years
Worker_8	22	Male	Construction	11 months
Worker_9	29	Male	Agriculture	3 years
Worker_10	34	Female	Manufacturing	4 years
Worker_11	38	Male	Manufacturing	7 years
Workers_12	34	Male	Welding	5 years
Worker_13	36	Female	Manufacturing	5 years
Worker_14	29	Male	Currently in shelter	4 years
Worker_15	33	Female	Plastic company	5 years
Worker_16	38	Male	Currently in shelter	5 years
Worker_17	39	Male	Furniture	6 years
Community leader and worker_1	38	Male	Construction	4 years
Community leader and worker_2	35	Male	Welding	5 years
Community leader and worker_3	41	Male	Manufacturing	7 years

## Data Availability

Data are available upon request from the corresponding author of the study.
